# Supplementary material for: Evolutionary Origin of MUTYH Germline Pathogenic Variations in Modern Humans
Source: Biomolecules. 2023 Feb 24;13(3):429. doi: 10.3390/biom13030429 (PMC10046817; doi:10.3390/biom13030429)
Supplement: Supplementary file 1 [file biomolecules-13-00429-s001.zip › Figures S1 and S2.pdf]

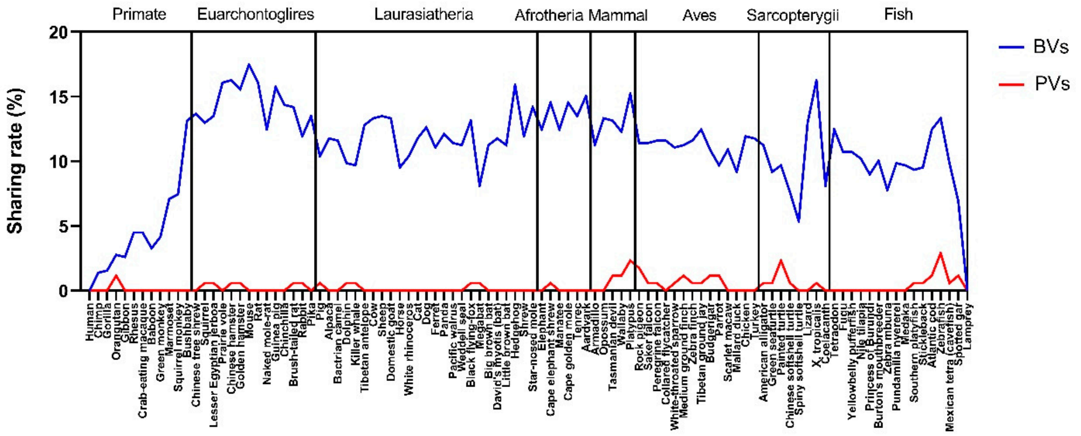

Figure S1. Quantitative distribution of human *MUTYH* PVs and BVs in 100 vertebrates.

The sharing rate (Y axis) of each species (X axis) was calculated by dividing the sharing number of variants by the total number of PVs or BVs. It showed that human *MUTYH* BVs were shared with most of the 100 vertebrate's species although the sharing rate in Primate was lower than in other clades, whereas human *MUTYH* PVs barely shared with other species. Red line: PVs.; Blue line: BVs. Independent-samples Mann-Whitney U test showed significant differences for the sharing rates between PVs and BVs ($P<0.05$).

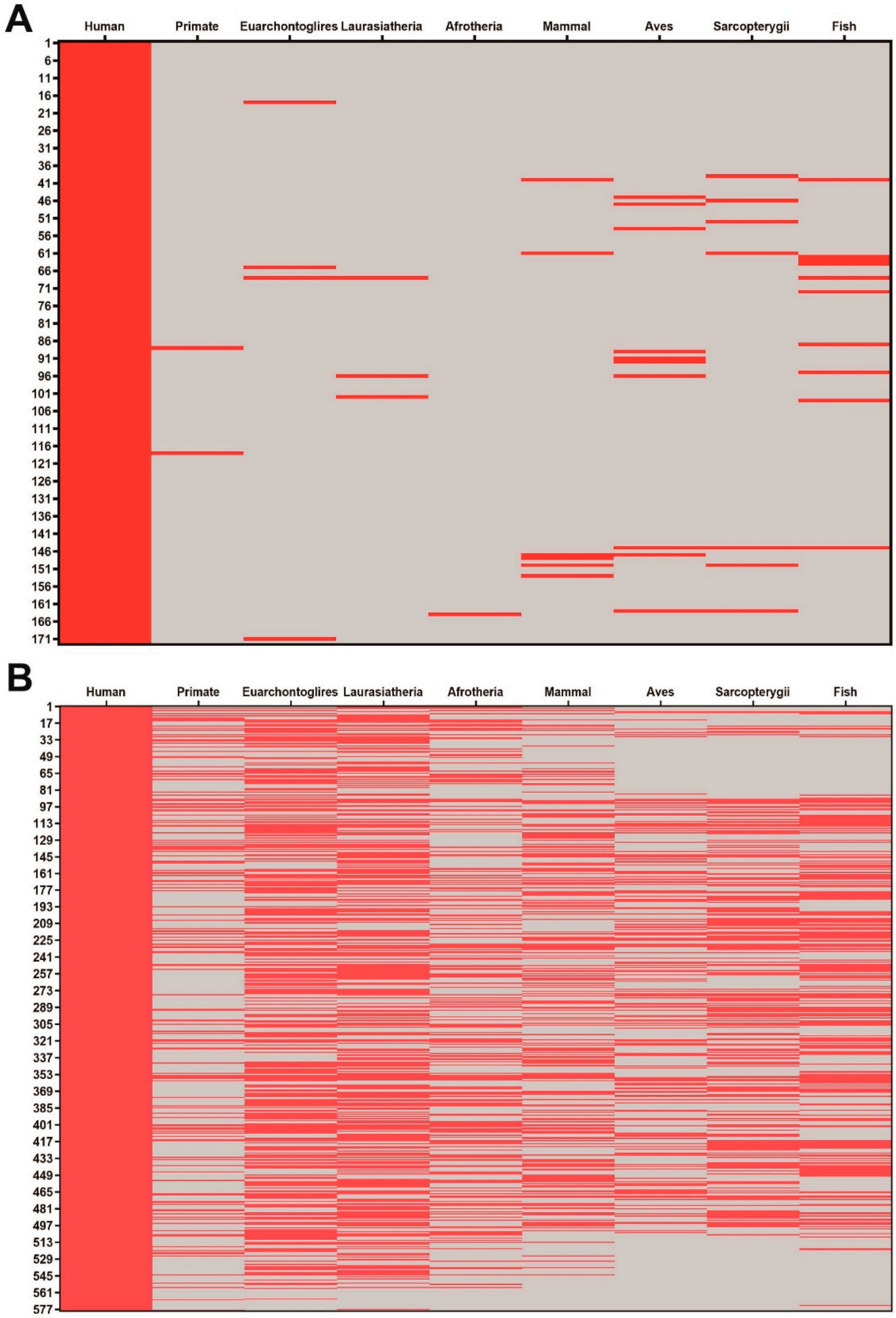

Figure S2. Distribution of different groups of human germline *MUTYH* variants in eight clades. A. PVs; B. BVs; Y-axis in A: number of 172 *MUTYH* PVs, Y-axis in B: number of 578 BVs; X-axis: the eight clades in the order from the closest to the most distant with the human at the left. Red in whole column: full set of PVs or BVs used in the study. Red cells: species in a clade shared at least 1 human variant, grey: no species in the clade shared human variant. continuous red line: species across different clades shared the same human variant.
